# Efficacy of Once-weekly Somatrogon in Children with Growth Hormone Deficiency After Switching from Daily Therapy

**DOI:** 10.2174/0118715303452569260114103416

**Published:** 2026-04-06

**Authors:** Cristina Giusto, Marina Passeri, Isabella Nardone, Sium Wolde Sellasie, Simona Zaccaria, Laura Giurato, Luigi Uccioli

**Affiliations:** 1 Division of Endocrinology and Diabetes, Department of Biomedicine and Prevention, CTO Andrea Alesini Hospital, University Tor Vergata, Rome 00133, Italy;; 2 School of Applied Medical-Surgical Sciences, University of Rome Tor Vergata, Rome 00133, Italy

**Keywords:** Growth hormone deficiency, pediatric, growth rate, daily therapy, weekly therapy, long-acting growth hormone, somatrogon

## Abstract

**Introduction:**

Long-acting Growth Hormone (LAGH) analogs represent a promising alternative to daily Growth Hormone (GH) therapy, aiming to improve adherence without compromising efficacy. Among them, somatrogon is the only LAGH currently available in Italy for pediatric use.

**Objective:**

The objective of this study is to evaluate growth outcomes and IGF-1 variability along with adherence in pediatric patients with Growth Hormone Deficiency (GHD) after switching from daily GH to weekly somatrogon.

**Methods:**

This retrospective, monocentric study involved 23 children and adolescents affected by GHD and in treatment with somatrogon for at least 12 months, after switching from daily therapy. We analyzed changes in growth velocity, serum IGF-1 and height velocity compared to target age-based references before and after the switch. To account for the age-related variability in our cohort, IGF-1 levels were then expressed as Standard Deviation Score (IGF-1 SDS), to quantify how much an individual value deviates from the age and sex-matched population mean. A questionnaire was administered to evaluate adherence and patients’ preferences.

**Results:**

Mean height velocity was slightly higher after the therapeutic switch (7.91 ± 1.7 cm/year *vs*. 7.12 ± 1.3 cm/year, *p* = 0.04), but no significant differences were observed when growth velocity was normalized to age-based targets or when comparing IGF-1 and IGF-1 SDS levels. Patients preferred the weekly therapy, even if missing doses and a few side effects were still reported.

**Discussion:**

Our findings suggest that somatrogon treatment is as effective as daily GH treatment in terms of growth velocity and IGF-1 levels in a pediatric population with growth hormone deficiency. Weekly therapy also seemed to be more manageable for the patients and their families.

**Conclusion:**

Weekly somatrogon maintains growth-promoting effects comparable to daily GH therapy in pediatric patients with GHD. Patients’ adherence and satisfaction contribute to making this therapy a valid alternative to daily treatments.

## 1 INTRODUCTION

Growth Hormone Deficiency (GHD) is a rare condition characterized by inadequate secretion of the Growth Hormone (GH) by the anterior pituitary gland. This clinical syndrome may occur in association with deficiencies of other pituitary hormones and can have congenital or acquired causes, but in children, it is generally isolated and idiopathic [1, 2]. A recent systematic review of mostly European studies reported a prevalence between 1/1107 and 1/8646, with an incidence ranging between 1/28,800 and 1/46,700 cases per year [3]. An Italian database registered in the Piedmont region between 2002 and 2004 reported a higher incidence (0.2/1000) and prevalence (1/1000) [3, 4].

The diagnosis of GHD requires a thorough medical history and physical examination, along with the assessment of anthropometric parameters over time, and should be suspected in case of deviation of the height from the target, height inferior to -2 SDS for age and sex, or deflection of growth rate with the loss of at least 0.3 SDS/year [5, 6]. Other causes of short stature must be excluded, and assessment of other pituitary hormones must be performed [5]. Isolated GH measurement is not useful in assessing deficiency due to pulsatile secretion. IGF-1 levels should be assessed and related to age [7]. Treatment with growth hormone begins after completing at least two positive diagnostic tests and continues until the end of longitudinal growth, at which point retesting is typically conducted with additional stimulation tests [1, 8, 9].

Traditional treatment involves the daily administration of recombinant GH formulations [10]. Despite its known efficiency, daily injections often lead to poor therapeutic adherence and frustration for patients and their families [11, 12]. Recently, long-acting molecules have been formulated, leading to weekly injections with the prospect of increased therapy adherence. Somatrogon is currently the only long-acting growth hormone administered weekly in Italy, designed for the treatment of growth hormone deficiency in children. It contains the amino-acid sequence of hGH and three copies of the C-terminal peptide of human chorionic gonadotropin, which increases its half-life [13, 14]. Suggested initial dose is 0.66 mg/Kg once weekly, with dose adjustments for efficacy and safety based on the measurement of IGF-1 4 days after the injection [15, 16].

Currently, a limited number of studies have demonstrated the non-inferiority of somatrogon treatment compared to daily GH in terms of efficacy, side effects, and therapeutic adherence. The primary objective of the study is to assess changes in growth velocity in patients receiving daily GH treatment after switching to weekly somatrogon therapy. The secondary objective is to evaluate any variations in IGF-1 levels, as well as the assessment of therapeutic adherence and side effects after the therapeutic switch.

## 2 MATERIALS AND METHODS

A retrospective, monocentric study was conducted, including 23 Italian patients with GHD, attending the Endocrinology Unit of the CTO Hospital of Rome between 2023 and 2025, 6 females and 17 males, with an average age of 13.7 years (range 11.4-16.5). All of them were being treated with somatrogon at an average dose of 0.60 mg/Kg/week (equivalent to 0.0126 µmol/kg/week), administered subcutaneously once a week. The dosage recommended by pivotal registration trials (0.66 mg/Kg/week) had been approximated for each patient to increase its manageability, considering the total dosage of the pen. All patients had previously received daily GH treatment at variable dosages between 0.13-0.27 mg/Kg/week (average dosage 0.17 mg/Kg/week, equivalent to 0.0077 µmol/kg/week), based on clinical and biochemical parameters. Given the retrospective design, no formal sample size calculation was performed. All patients meeting the inclusion criteria and with complete data available were included. Inclusion criteria were as follows: diagnosis of GH deficiency, age over 3 years and under 18 years, treatment with somatrogon, and previous treatment with daily GH for at least 6 months. Patients at the end of longitudinal growth and patients who did not comply with the informed consent were excluded. Clinical and laboratory data were collected from medical reports before and 12 months after switching to long-acting GH. For each patient, growth rate (cm/year) and IGF-1 (ng/ml) levels before and after 12 months from the therapeutic switch were considered. Tanner growth charts were used to assess growth velocity percentiles for age and sex before and after the switch. IGF-1 was assessed the morning after the injection for daily GH and 4 days after the injection of long-acting GH. To eliminate age and sex related variability, IGF-1 levels were then converted to IGF-1 SDS using the appropriate age and sex-matched normative data corresponding to the assay method used by our laboratory. The rationale for its use lies in the fact that serum IGF-1 levels vary significantly during childhood and adolescence. Therefore, absolute values (ng/mL) are not directly comparable between patients of different ages or in the same patient over time. The use of SDS allows for data standardization, enabling an accurate assessment of the adequacy of the treatment and ensuring that levels remain within the safe therapeutic range (between -2 and +2 SDS). The manageability of both therapies was evaluated through a scale from 1 to 5, where 1 was not at all manageable and 5 was very manageable. The therapeutic adherence was evaluated through a question about missed doses in the last month, while side effects were assessed through open-ended questions. Patients and caregivers were also asked whether they preferred the current weekly therapy or the previous daily therapy and if they felt freer in their daily life with the new therapy.

## 3 RESULTS

### 3.1 Growth Velocity

Annual growth velocity was slightly higher after switching to weekly treatment: 7.12 ± 1.3 cm/year before *vs*. 7.91 ± 1.7 cm/year after the switch (*p* = 0.04).

### 3.2 Comparison between Growth Velocity Percentiles

**Fig. (1):**
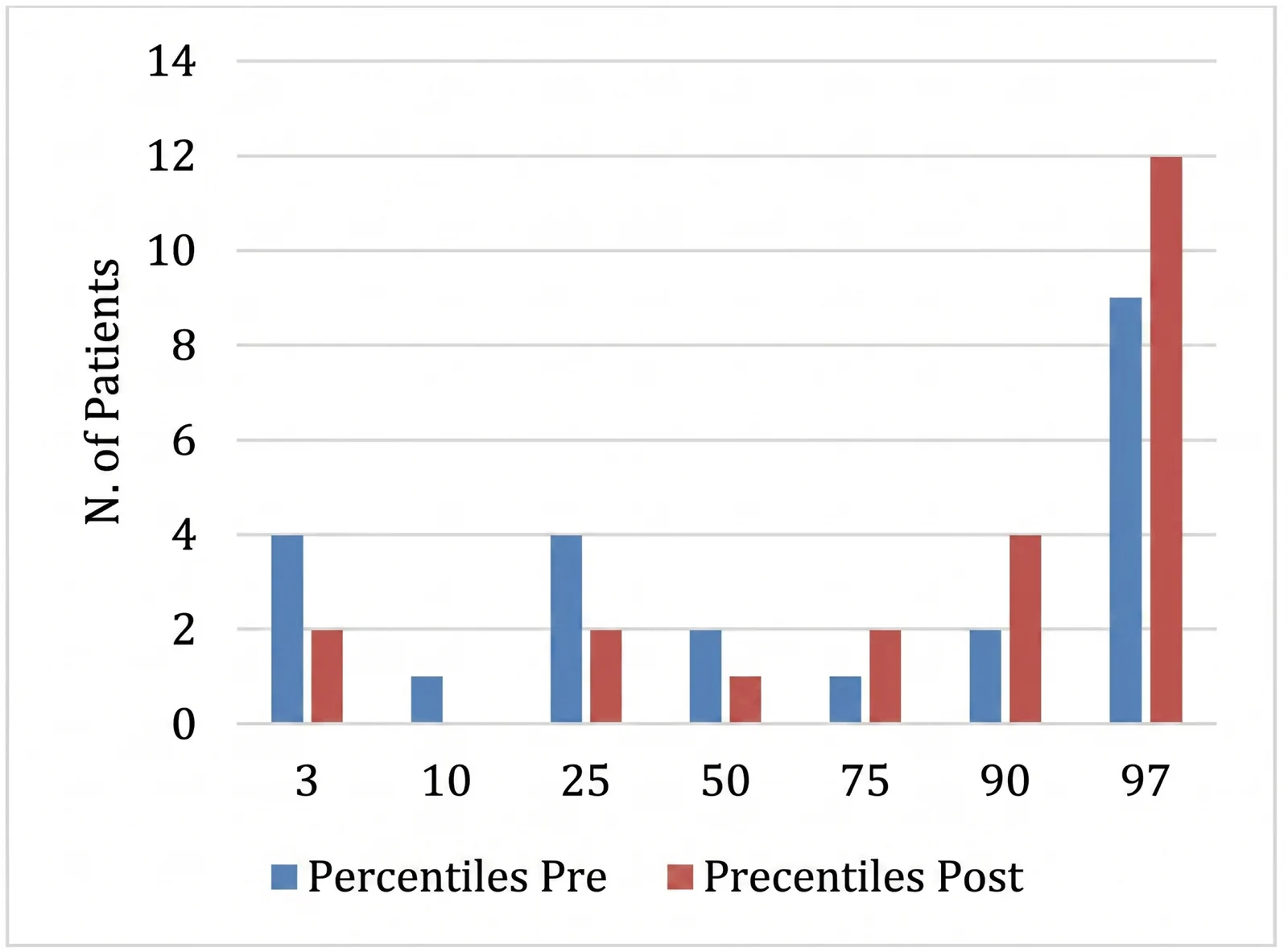
Frequency distribution of growth velocity percentiles before and 12 months after the therapeutic switch.

**Fig (2):**
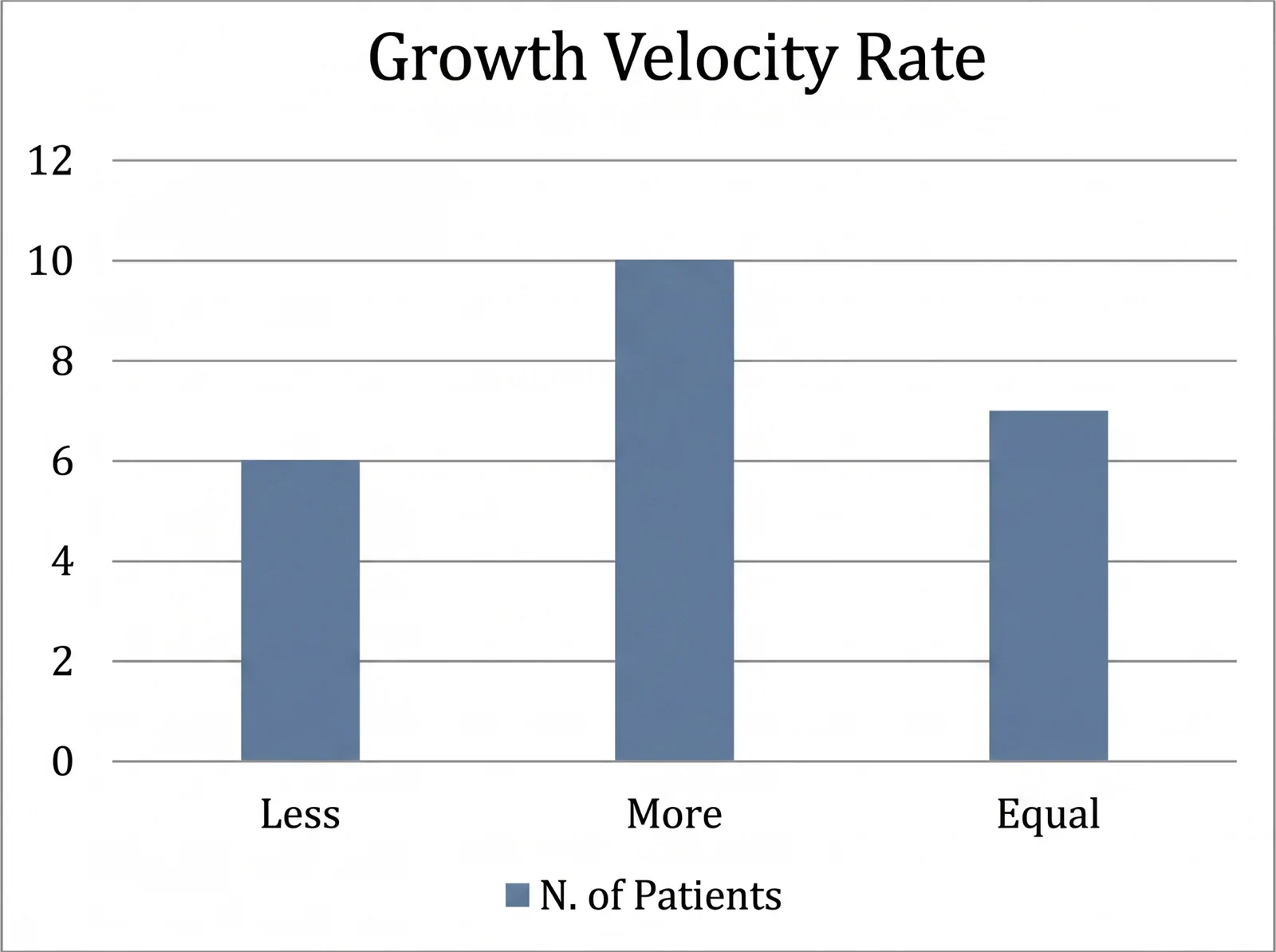
Distribution of variations in growth velocity percentiles.

The comparison between growth velocity percentiles before and 12 months after the treatment change showed no significant differences in frequency distributions (*p* = 0.23) (Figs. **1** and **2**).

### 3.3 IGF-1

**Fig. (3):**
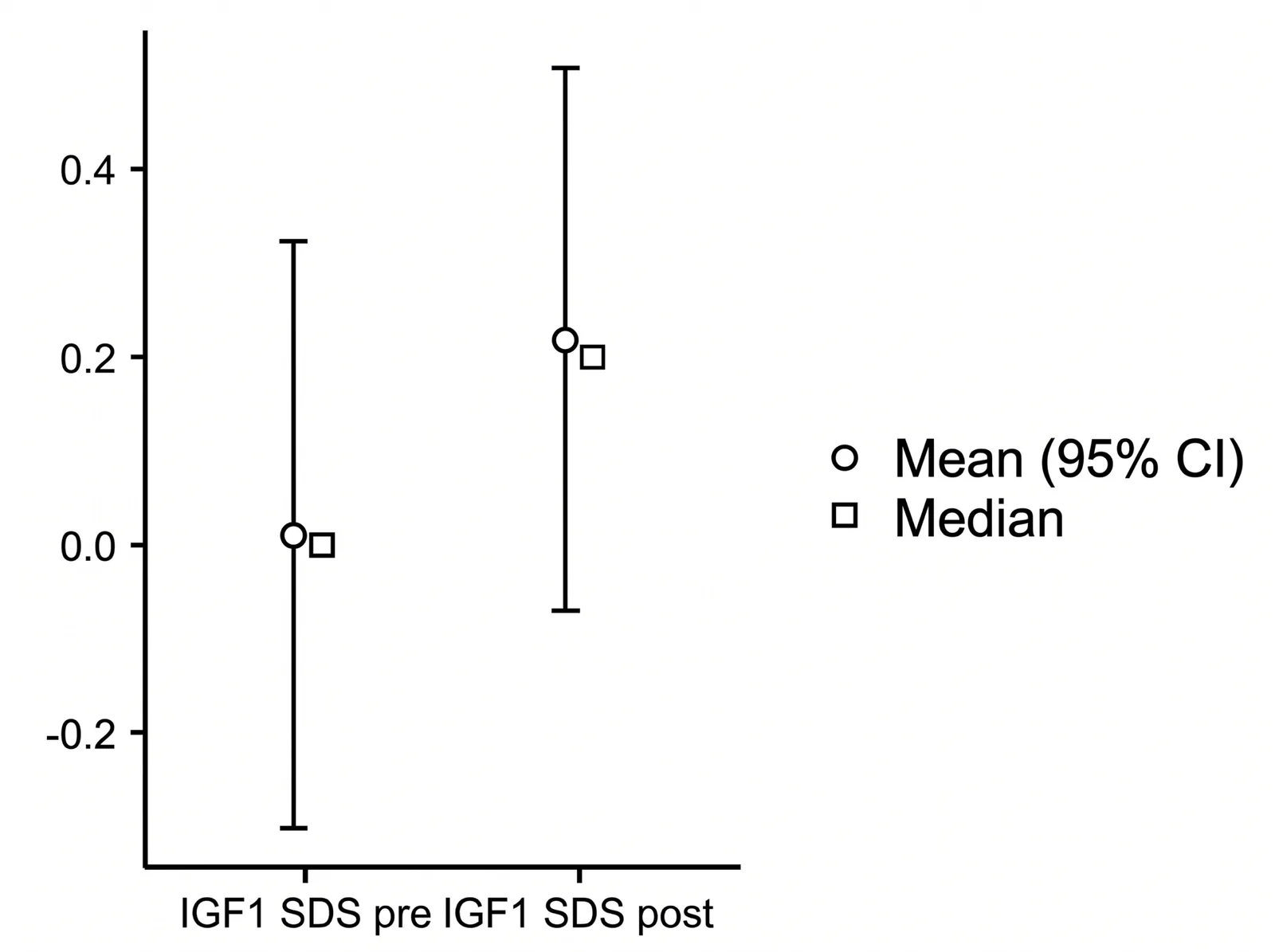
IGF-1 SDS before and after the switch.

An increase in IGF-1 levels was observed after switching to somatrogon: 341.6 ± 106 ng/ml *vs* 401.1 ± 116 ng/ml. Nevertheless, it was not statistically significant (*p* = 0.08). Furthermore, mean IGF-1 SDS levels were 0.0 ± 0.7 before and 0.2 ± 0.7 after the therapeutic switch (Fig. **3**). The comparison between the two periods did not show statistically significant differences (*p* = 0.31).

### 3.4 Manageability, Therapeutic Adherence, and Side Effects

On a scale of 1 to 5, the mean score given by patients for the manageability of weekly GH therapy was 4.8, compared to a mean score of 3.3 given for the manageability of previous daily GH therapy. The totality of patients reported that they preferred weekly therapy to daily therapy and felt freer in their activities with weekly therapy. Regarding adherence, 7 of the 23 patients (30%) reported one missed dose in the previous month, due in three cases to forgetting to administer the medication, in two cases to practical difficulties in bringing the therapy on vacation, and in the last two cases to forgetting to order the therapy. Three patients reported that the weekly GH injection was more painful than the previous daily formulation, and one patient reported headache and knee pain the day of the injections.

## 4 DISCUSSION

This retrospective study evaluated the clinical and biochemical efficacy of once-weekly somatrogon in a pediatric population with growth hormone deficiency after switching from daily GH therapy. After 12 months of long-acting therapy, growth velocity showed a slight but statistically significant increase, while IGF-1 levels remained stable, with no significant variation. The observed increase in annual growth velocity (7.91 *vs*. 7.12 cm/year) suggests that weekly somatrogon therapy is at least as effective as daily GH and may, in some cases, offer enhanced auxological outcomes. However, when growth velocity was normalized to age- and sex-specific reference values, no significant difference was observed in the percentiles distribution before or after the switch. This indicates that, although there is a modest increase in growth velocity in absolute terms, the effectiveness of weekly GH treatment is comparable to that of daily GH when adjusted for sex and age-specific target.

These findings are consistent with data from pivotal trials and real-world studies on somatrogon, which have demonstrated non-inferiority to daily GH in terms of both linear growth and IGF-1 SDS levels [13, 17, 18].

The lack of significant IGF-1 and IGF-1 SDS variation post-switch confirms stable GH/IGF-1 axis activation, likely due to the sustained pharmacokinetic profile of somatrogon, and indicates maintenance of biochemical control [19].

From a clinical perspective, reduced injection frequency may improve treatment adherence, particularly in adolescents. In our experience, weekly therapy seemed to be more manageable for the patients and their families, who reported feeling freer in their daily lives. Despite that, missing doses were still reported.

Three patients reported that the injection was more painful, which might be explained by the increased viscosity of the weekly formulation [20, 21]. However, there were no cases of discontinuation of the therapy.

## 5 LIMITATIONS OF THIS STUDY

The main limitation of this study is the short follow-up duration. Although growth velocity was maintained or improved after the switch, such a short period does not allow us to evaluate the impact of somatrogon on final height. Current data on somatrogon suggest non-inferiority to daily GH, but long-term follow-up, until final height is reached, is needed to confirm whether the results in terms of adult height are comparable to those obtained with standard therapy.

Other limitations include the relatively small sample size and non-randomized design. Moreover, the population was predominantly pubertal, a developmental stage characterized by hormonal changes that may independently influence growth velocity. Nonetheless, the use of growth velocity percentiles and IGF-1 SDS and the uniform use of a single long-acting GH molecule strengthen the validity of the findings.

## CONCLUSION

Our study provides real-world evidence on the use of long-acting GH in children with growth hormone deficiency. Switching from daily GH to once-weekly somatrogon in children and adolescents with GHD was associated with stable IGF-1 SDS levels and age-related growth velocity. Moreover, the new therapy was associated with good treatment acceptance and a high level of satisfaction, both from patients and caregivers. These data support the use of long-acting GH formulations as clinically effective alternatives to daily therapy, particularly in populations where reducing treatment burden may enhance long-term adherence. Further long-term studies with larger cohorts are needed to confirm these results and to better define the long-term effects of the long-active treatment.

## Data Availability

All data generated or analyzed during this study are included in this published article.

## LIST OF ABBREVIATIONS

LAGH: Long-acting Growth Hormone
GH: Growth Hormone
GHD: Growth Hormone Deficiency
IGF-1: Insulin-like Growth Factor 1
IGF-1 SDS: Insulin-like Growth Factor 1 Standard Deviation Score

## STANDARDS OF REPORTING

STROBE guidelines were followed.
